# A novel autophagy‐related lncRNA survival model for lung adenocarcinoma

**DOI:** 10.1111/jcmm.16582

**Published:** 2021-05-13

**Authors:** Liwei Wu, Zilu Wen, Yanzheng Song, Lin Wang

**Affiliations:** ^1^ Department of Thoracic Surgery Shanghai Public Health Clinical Center Fudan University Shanghai China; ^2^ Department of Scientific Research Shanghai Public Health Clinical Center Fudan University Shanghai China; ^3^ TB Center Shanghai Emerging & Re‐emerging Infectious Diseases Institute Shanghai China

**Keywords:** autophagy, long non‐coding RNA (lncRNA), lung adenocarcinoma (LUAD), survival, The Cancer Genome Atlas (TCGA)

## Abstract

Long non‐coding RNA (lncRNA) is an important regulatory factor in the development of lung adenocarcinoma, which is related to the control of autophagy. LncRNA can also be used as a biomarker of prognosis in patients with lung adenocarcinoma. Therefore, it is important to determine the prognostic value of autophagy‐related lncRNA in lung adenocarcinoma. In this study, autophagy‐related mRNAs‐lncRNAs were screened from lung adenocarcinoma and a co‐expression network of autophagy‐related mRNAs‐lncRNAs was constructed by using The Cancer Genome Atlas (TCGA). The univariate and multivariate Cox proportional hazard analyses were used to evaluate the prognostic value of the autophagy‐related lncRNAs and finally obtained a survival model composed of 11 autophagy‐related lncRNAs. Through Kaplan‐Meier analysis, univariate and multivariate Cox regression analysis and time‐dependent receiver operating characteristic (ROC) curve analysis, it was further verified that the survival model was a new independent prognostic factor for patients with lung adenocarcinoma. In addition, based on the survival model, gene set enrichment analysis (GSEA) was used to illustrate the function of genes in low‐risk and high‐risk groups. These 11 lncRNAs were GAS6‐AS1, AC106047.1, AC010980.2, AL034397.3, NKILA, AL606489.1, HLA‐DQB1‐AS1, LINC01116, LINC01806, FAM83A‐AS1 and AC090559.1. The hazard ratio (HR) of the risk score was 1.256 (1.196‐1.320) (*P* < .001) in univariate Cox regression analysis and 1.215 (1.149‐1.286) (*P* < .001) in multivariate Cox regression analysis. And the AUC value of the risk score was 0.809. The 11 autophagy‐related lncRNA survival models had important predictive value for the prognosis of lung adenocarcinoma and may become clinical autophagy‐related therapeutic targets.

## 1 INTRODUCTION

Lung cancer is the cancer with the highest morbidity and mortality among cancers.^1^ Lung adenocarcinoma (LUAD) is the most common pathological subtype of lung cancer, accounting for 45% of all lung cancer.^2^ Despite the continuous progress in the technology of cancer diagnosis and treatment, the mortality rate of lung cancer remains high. One reason is that some patients are diagnosed with advanced lung cancer, and the other is that the existing guided staging system is not accurate in predicting the prognosis of lung cancer; as a result, some patients with early lung cancer did not receive adjuvant therapy after operation, which led to the recurrence or metastasis of lung cancer.^3^, ^4^ Therefore, it is necessary to update the staging system of the existing guidelines.

Autophagy is a highly conservative physiological process, which maintains the stability of the intracellular environment through the lysosome degradation system.^5^, ^6^ Autophagy plays an important role in many physiological processes, such as immune response, inflammation and tumorigenesis.^7^, ^8^ In the past few decades, there have been more and more studies on autophagy in LUAD.^9^, ^10^ Therefore, it is very important to establish an autophagy‐related gene set to predict the prognosis of LUAD.

Long non‐coding RNA (lncRNA) is a series of nucleotides with a length of more than 200 bp and does not have the ability to encode proteins.^11^ LncRNA is involved in many steps in the process of cancer occurrence and development, so it may be used as a biomarker to predict the prognosis of cancer patients.^12^, ^13^, ^14^ In addition, more and more studies have shown that in many cancers, lncRNA can promote the occurrence and development of tumours.^15^, ^16^, ^17^ Therefore, it is very important to screen the autophagy lncRNA related to the prognosis of LUAD.

In this study, a data set of gene expression in LUAD from The Cancer Genome Atlas (TCGA) was analysed and autophagy‐related lncRNA was screened out. The autophagy‐related lncRNA signature was identified to predict the survival prognosis of LUAD patients.

## 2 METHODS AND MATERIALS

### 2.1 LUAD patient data

The clinical data and gene expression data of patients with LUAD were collected from TCGA database (https://cancergenome.nih.gov/). Among them, the gene expression data used the data that had been normalized. In this study, the data of 954 patients with LUAD were analysed. Excluding patients with duplicated and missing clinical information, a total of 316 patient data were used for follow‐up analysis.

### 2.2 Screening of autophagy‐related lncRNA in LUAD

A list of genes related to autophagy was obtained from the Human Autophagy Database (http://www.autophagy.lu/). A total of 210 autophagy‐related genes were obtained from the LUAD gene expression data. Finally, 1651 autophagy‐related lncRNAs were screened by constructing autophagy‐related mRNA‐lncRNA co‐expression network according to the following criteria: |Correlation Coefficient| > 0.4 and *P* < .001.^18^ We used Pearson correlation analysis to perform the above analysis by limma R package.

### 2.3 Identification of autophagy‐related lncRNA prognostic signatures for LUAD

In order to identify the autophagy‐related lncRNA associated with survival, we conducted a univariate Cox proportional hazard analysis and Kaplan‐Meier analysis, and *P* < .01 was considered to be statistically significant. Then, the survival R package was used for multivariate Cox proportional hazard analysis, the optimal prognostic risk model was established, and the risk score was calculated by the following formula.

$$\text{Risk}\;\text{score}=\text{coef}\left(\text{lncRNA1}\right)\times \text{expr}\left(\text{lncRNA}1\right)+\text{coef}\left(\text{lncRNA}2\right)\times \text{expr}\left(\text{lncRNA}2\right)+\ldots +\text{coef}\left(\text{lncRNAn}\right)\times \text{expr}\left(\text{lncRNAn}\right).$$

$$\text{coef}\left(\text{lncRNAn}\right)\text{was}\;\text{defined}\;\text{as}\;\text{the}\;\text{coefficient}\;\text{of}\;\text{lncRNAs}\;\text{correlated}\;\text{with}\;\text{survival}.$$

$$\text{expr}\left(\text{lncRNAn}\right)\text{was}\;\text{defined}\;\text{as}\;\text{the}\;\text{expression}\;\text{of}\;\text{lncRNAs}.$$

The LUAD patients were divided into two groups by the median risk score: high‐risk group and low‐risk group. We performed Kaplan‐Meier survival analysis to estimate the survival difference between the two groups by using the survival R packages.

### 2.4 ROC curve plotting and independent prognostic analysis

Univariate and multivariate Cox analyses were performed to evaluate relationship of survival prognosis with clinical factors and risk score by using the survival R package. In order to estimate the predictive accuracy for survival time by different clinical factors and risk score, time‐dependent receiver operating characteristic (ROC) curves were plotted by using the survivalROC R package.

### 2.5 Construction and calibration of nomogram

The R package rms was used to construct nomogram which contained risk scores and clinical factors such as age, gender and stage. The nomogram can be used to predict the probable 3‐year and 5‐year survival of LUAD patients. The R package survival was utilized to plot calibration curve of nomogram. The calibration curve can intuitively demonstrate prediction ability of nomogram.

### 2.6 Statistical analysis

We used R software (version 3.6.2) to perform all statistical analyses. The Sankey diagram and Cytoscape software were used to visualize prognostic autophagy‐associated lncRNA‐mRNA co‐expression network. The functional annotation was performed by using gene set enrichment analysis (GSEA, https://www.gsea‐msigdb.org/gsea/index.jsp). GSEA is an important gene annotation tool,^19^ which can analyse and annotate the whole genetic data, thus avoiding the omission of key information. The *P* < .05 was considered to be statistically significant.

## 3 RESULTS

### 3.1 Autophagy‐related lncRNA with significant prognostic value in LUAD

**FIGURE 1:**
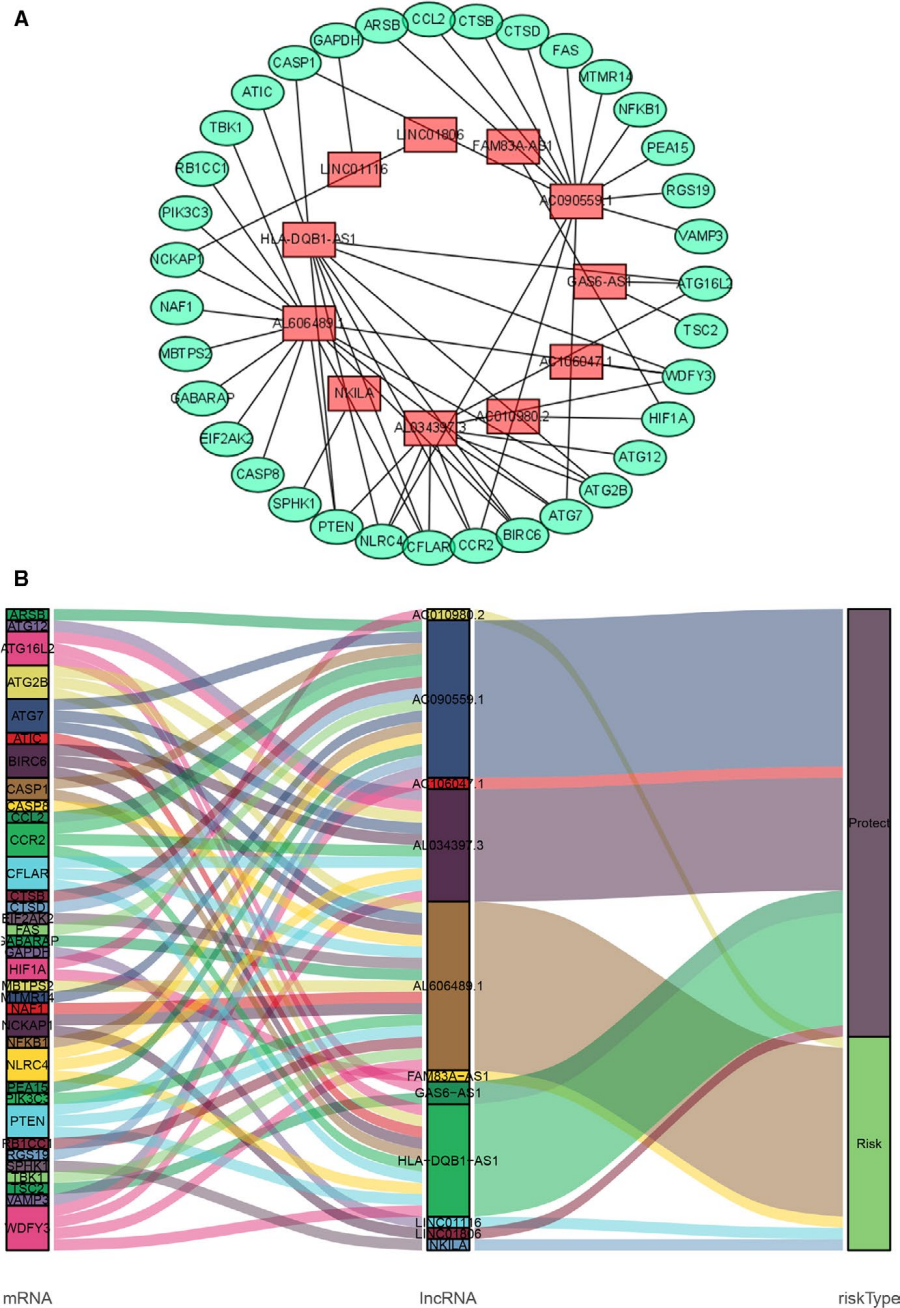
Screening of prognostic autophagy‐related lncRNA in LUAD. A, A prognostic co‐expression network of the 11 autophagy‐related lncRNAs‐mRNAs. B, The Sankey diagram of the relationship between lncRNA and mRNA

**FIGURE 2:**
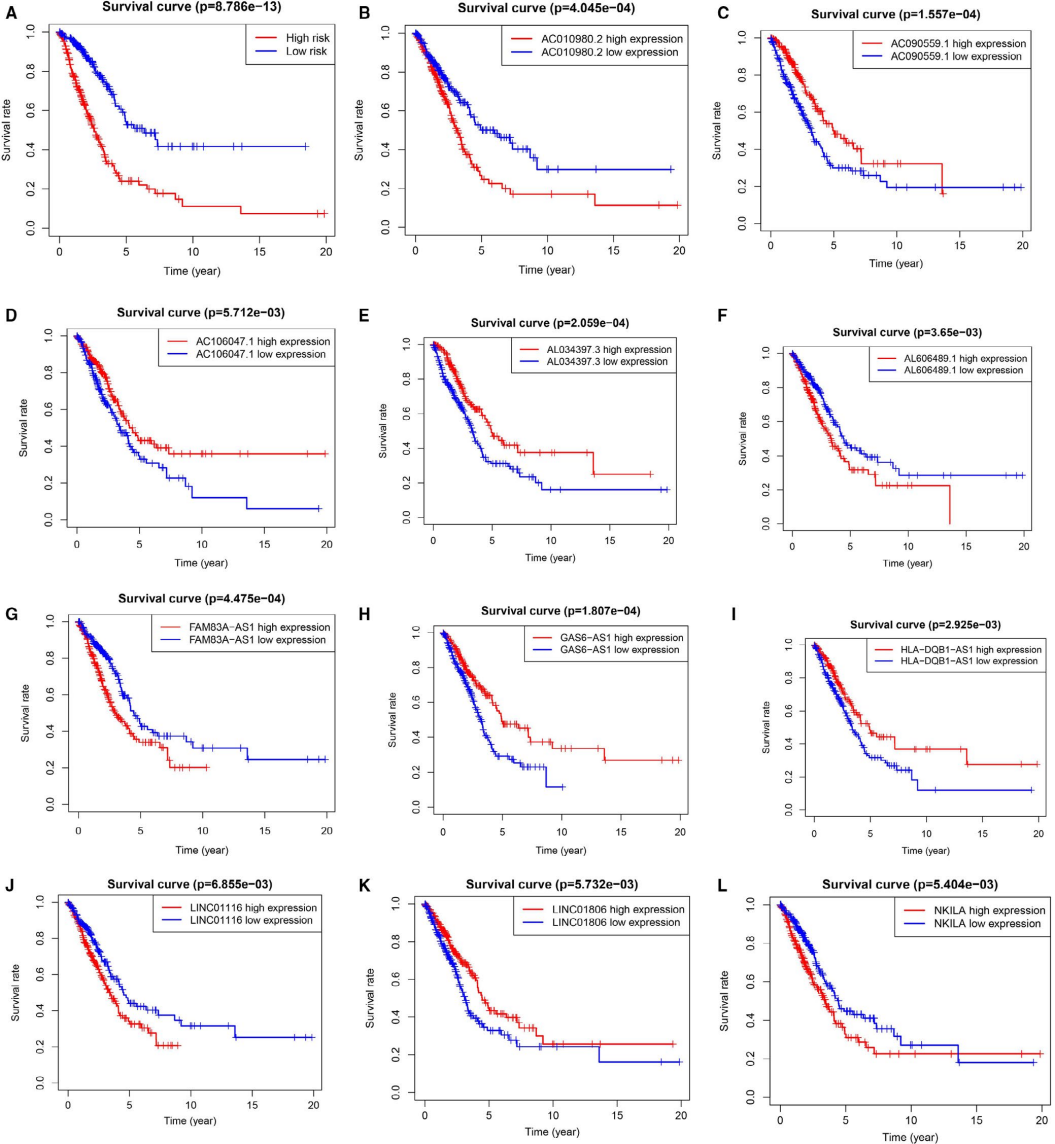
Survival curve of patients with LUAD in different groups

**FIGURE 3:**
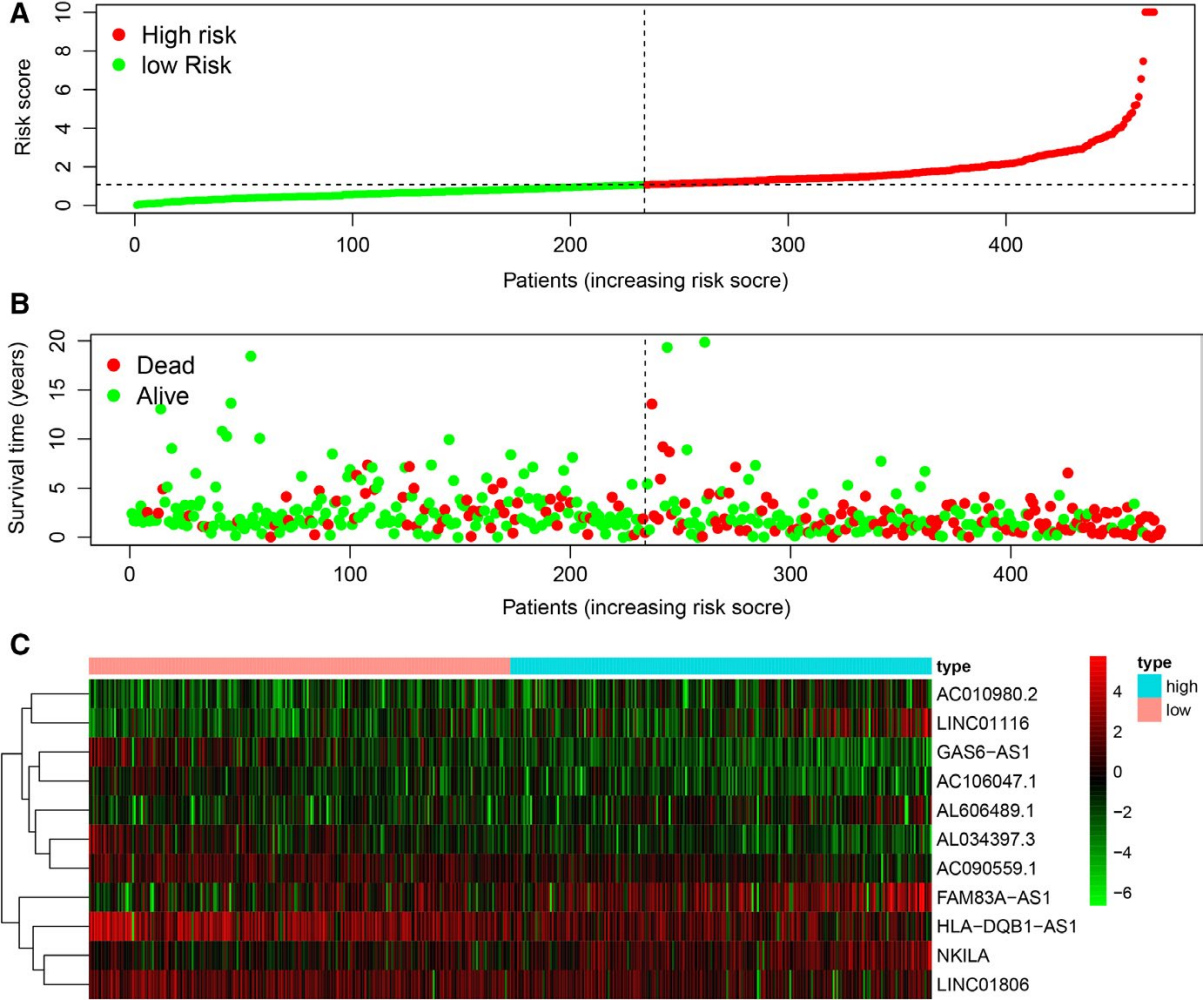
The survival model of the 11 autophagy‐related lncRNAs with prognostic value. A, The risk curve based on the risk score of each sample. B, The scatterplot based on the survival status of each sample. The green and red dots represent survival and death. C, The heatmap displayed the expression levels of autophagy‐related lncRNA in the high‐risk and low‐risk groups

Through the construction of autophagy‐related mRNA and lncRNA co‐expression network, a total of 1651 autophagy‐related lncRNAs were obtained. Among them, Cox proportional hazards analysis and Kaplan‐Meier analysis demonstrated that 33 autophagy‐related lncRNAs were significantly associated with the survival of LUAD patients from the TCGA (*P* < .01), including 23 lncRNAs with low risk (hazard ratio (HR)<1) and 10 lncRNAs with high risk (hazard ratio (HR)>1) (Table 1). Furthermore, multivariate Cox analysis screened 11 lncRNAs from the above 33 autophagy‐related lncRNAs with prognostic significance, and the names of 11 lncRNAs were GAS6‐AS1, AC106047.1, AC010980.2, AL034397.3, NKILA, AL606489.1, HLA‐DQB1‐AS1, LINC01116, LINC01806, FAM83A‐AS1 and AC090559.1 (Table 2). We used these 11 lncRNAs to establish the optimal prognostic risk model and established a prognostic visual co‐expression network of autophagy‐related lncRNA‐mRNA (Figure 1). According to the risk score formula, based on calculated median risk score, LUAD patients were divided into two groups: high‐risk group and low‐risk group. Meanwhile, according to the expression of 11 different lncRNAs, based on the calculated median expression, LUAD patients were divided into two groups: high expression group and low expression group. Kaplan‐Meier survival analysis showed that the overall survival (OS) of the high‐risk group was worse than that of the low‐risk group, indicating that risk score could predict the prognosis (Figure 2A). Similarly, the overall survival (OS) of the high expression group with high‐risk lncRNA was worse than that of the group with low expression (Figure 2B‐L). The risk score and the relevant survival statuses of LUAD patients were visualized by risk curve and scatterplot (Figure 3A,B), which demonstrated that the mortality occurrence depended on the risk score. The heatmap of these 11 autophagy‐related lncRNAs demonstrated that AC010980.2, NKILA, AL606489.1, LINC01116 and FAM83A‐AS1 were up‐regulated in the high‐risk group, while GAS6‐AS1, AC106047.1, AL034397.3, HLA‐DQB1‐AS1, LINC01806 and AC090559.1 were highly expressed in the low‐risk group (Figure 3C).

**TABLE 1:** LncRNA univariate Cox regression analyses and KM analysis of OS in lung adenocarcinoma patients

lncRNA	KM analysis	Univariate Cox regression analyses			
	KM *P*‐value	HR	HR 95 low	HR 95 high	*P*‐value
GAS6‐AS1	.0002	0.7767	0.6512	0.9265	.0050
UGDH‐AS1	.0008	0.5991	0.4357	0.8236	.0016
MMP2‐AS1	.0066	0.7832	0.6514	0.9418	.0094
AC106047.1	.0057	0.6982	0.5362	0.9091	.0076
AC010980.2	.0004	1.2306	1.0620	1.4260	.0058
AC099850.3	.0092	1.0337	1.0112	1.0568	.0031
AC245595.1	.0002	1.3306	1.1858	1.4931	.0000
AC021016.2	.0035	0.6311	0.4617	0.8626	.0039
CARD8‐AS1	.0095	0.8285	0.7184	0.9555	.0097
RPARP‐AS1	.0030	0.8386	0.7471	0.9414	.0028
AC090559.1	.0002	0.7948	0.6840	0.9235	.0027
AL034397.3	.0002	0.7526	0.6220	0.9106	.0035
AC011477.2	.0001	0.8287	0.7350	0.9344	.0022
AL691432.2	.0002	0.8315	0.7520	0.9195	.0003
CRNDE	.0005	0.9521	0.9249	0.9801	.0009
AC008764.2	.0073	0.8452	0.7602	0.9397	.0019
NKILA	.0054	1.1095	1.0578	1.1638	.0000
VIM‐AS1	.0009	0.8167	0.7263	0.9184	.0007
AC019069.1	.0021	1.2033	1.0699	1.3533	.0020
ITGB1‐DT	.0007	1.0427	1.0132	1.0730	.0043
AL161785.1	.0011	0.8964	0.8316	0.9663	.0043
MGC32805	.0088	0.8193	0.7092	0.9465	.0068
AL606489.1	.0037	1.2531	1.1198	1.4024	.0001
AL137003.1	.0023	0.7441	0.5978	0.9263	.0082
AC006449.6	.0077	0.7465	0.6010	0.9271	.0082
AL365203.2	.0002	1.2307	1.1373	1.3318	.0000
HLA‐DQB1‐AS1	.0029	0.9243	0.8881	0.9621	.0001
LINC01116	.0069	1.1063	1.0718	1.1419	.0000
LINC01806	.0057	0.8766	0.7987	0.9620	.0055
LINC00324	.0022	0.7842	0.6580	0.9346	.0066
FAM83A‐AS1	.0004	1.0469	1.0279	1.0661	.0000
AC087752.3	.0005	0.6885	0.5256	0.9020	.0068
AL035587.1	.0044	0.6665	0.5015	0.8856	.0052

**TABLE 2:** LncRNA multivariate Cox regression analyses of OS in lung adenocarcinoma patients

lncRNA	Coef	HR	*P*‐value	PH *P*‐value
GAS6‐AS1	−0.141	0.868	.000	.311
AC106047.1	−0.280	0.756	.003	.209
AC010980.2	0.181	1.199	.006	.113
AL034397.3	−0.186	0.830	.008	.024
NKILA	0.080	1.083	.005	.276
AL606489.1	0.167	1.182	.000	.517
HLA‐DQB1‐AS1	−0.043	0.958	.000	.395
LINC01116	0.061	1.063	.000	.989
LINC01806	−0.167	0.846	.006	.617
FAM83A‐AS1	0.031	1.031	.000	.127
AC090559.1	−0.142	0.868	.003	.338

Note

Global PH *P*‐value was .105; thus, the Cox regression model can be used to construct the predictive model.

### 3.2 Evaluation of the survival model for LUAD patients

**FIGURE 4:**
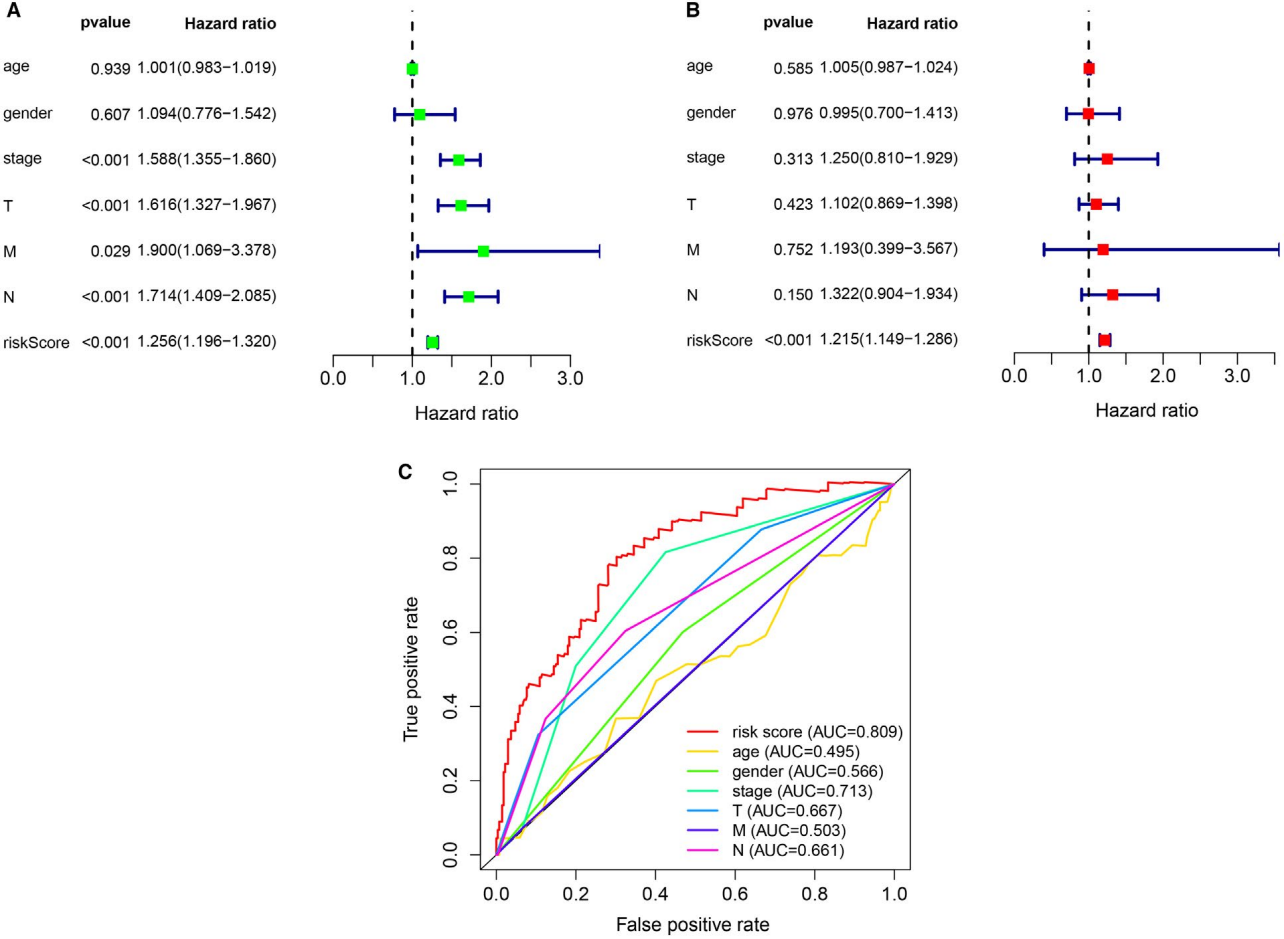
Assessment of the prognostic survival model of the 11 autophagy‐related lncRNAs in LUAD. A, The results of univariate Cox regression analysis of risk score and clinical factors. B, The results of multivariate Cox regression analysis of risk score and clinical factors. C, The AUC for risk score and clinical factors and the ROC curves. Clinical factors: age, gender, stage, T (tumour size), N (lymph node metastasis) and M (distant metastasis)

**FIGURE 5:**
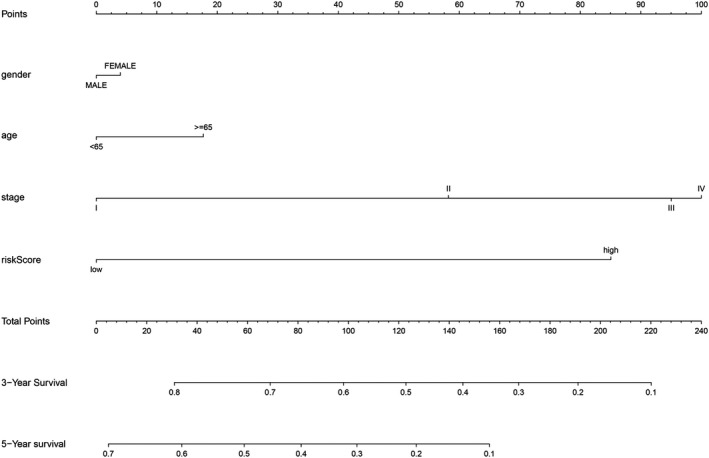
The nomogram of risk score and clinical factors

**FIGURE 6:**
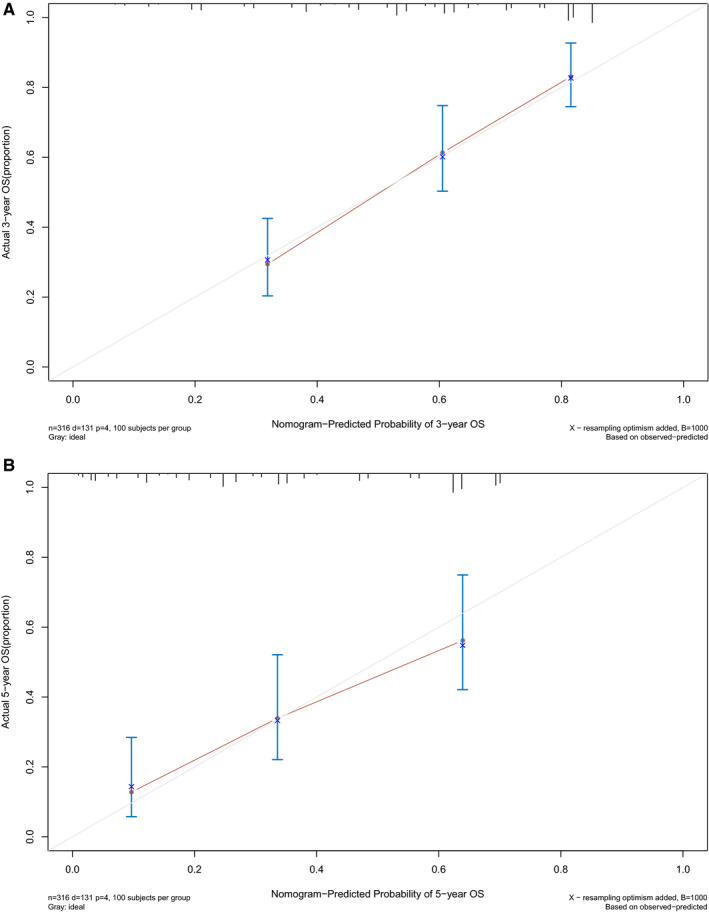
The calibration curve of nomogram. A, The 3‐year OS calibration curve. B, The 5‐year OS calibration curve. The closer the red solid line is to the grey solid line, the closer the nomogram prediction probability is to the actual probability

In order to evaluate whether the above 11 autophagy‐related lncRNA survival models were independent prognostic factors of LUAD, univariate and multivariate Cox regression analyses were performed. The hazard ratio (HR) of the risk score was 1.256 (95% CI 1.196‐1.320) (*P* < .001) in univariate Cox regression analysis (Figure 4A) and 1.215 (95% CI 1.149‐1.286) (*P* < .001) in multivariate Cox regression analysis (Figure 4B). Therefore, the 11 autophagy‐related lncRNAs were independent prognostic factors for LUAD. The area under the ROC curve of risk score (AUC) was calculated to evaluate the sensitivity and specificity of risk score in predicting the prognosis of patients with LUAD. The AUC value of the risk score was 0.809, which was higher than that of other clinical factors (Figure 4C), indicating that 11 autophagy‐related lncRNAs were quite reliable for the prognostic risk model of LUAD. A nomogram plot was performed to predict 3‐year and 5‐year survival in LUAD patients by using age, gender, stage and risk score (Figure 5). The calibration curve showed the good prediction ability of nomogram, and the C‐index of nomogram was 0.746 (Figure 6).

### 3.3 GSEA enrichment

**FIGURE 7:**
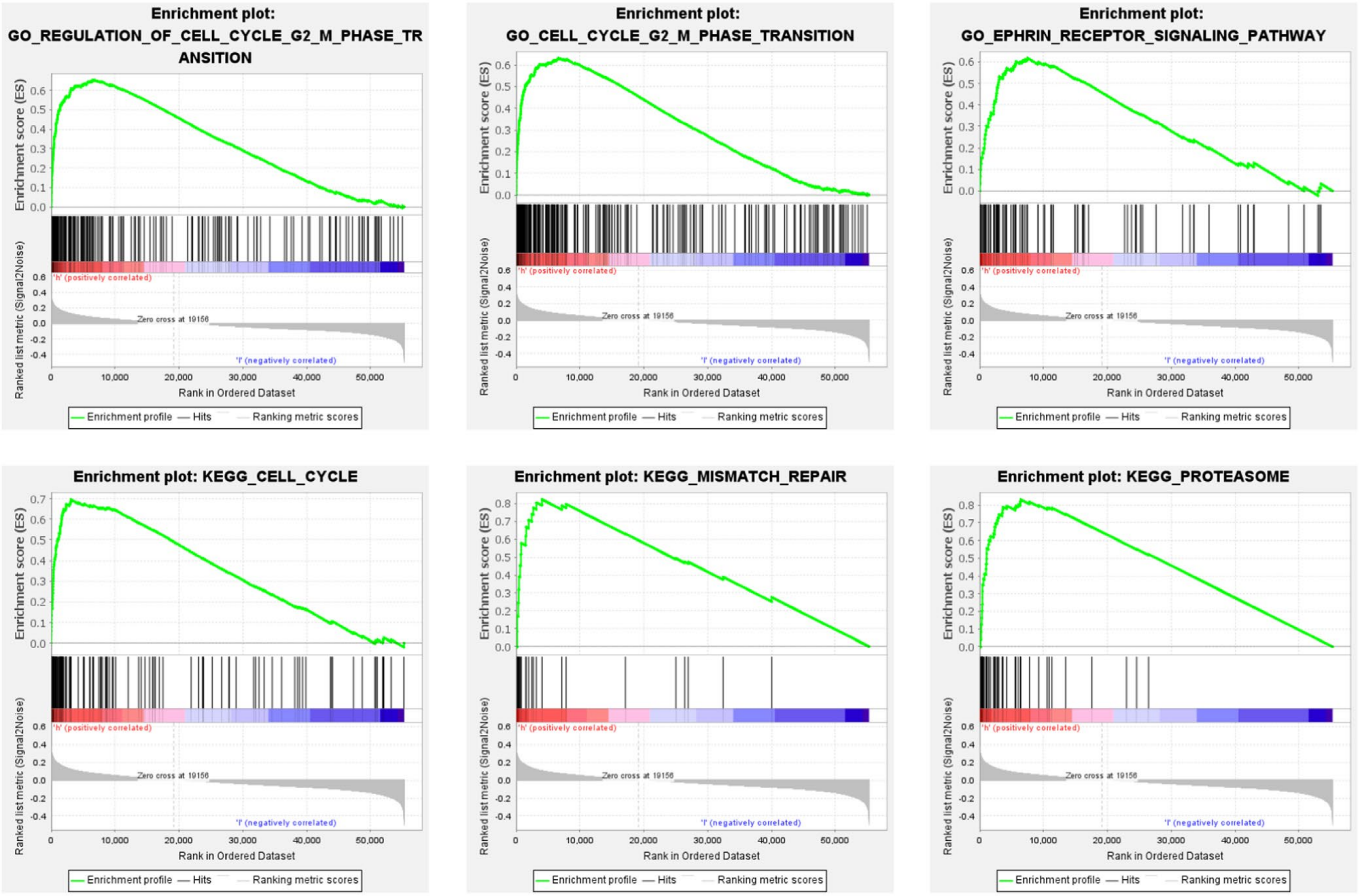
Gene Ontology (GO) and KEGG analyses of the 11 autophagy‐related lncRNAs by GSEA

GSEA showed that there were different gene expression patterns between high‐risk group and low‐risk group. In the high‐risk group, the expression of genes related to cell cycle and mismatch repair was higher, while in the low‐risk group, the expression of genes related to cell cycle mismatch repair was lower (Figure 7).

## 4 DISCUSSION

In the field of cancer treatment, the treatment of LUAD tends to be individualized and accurate.^20^, ^21^ However, according to the current guidelines for cancer diagnosis and treatment, it is difficult to apply individualized and accurate treatment. According to clinical experience, some patients with early LUAD will still have tumour recurrence and metastasis after surgical treatment, and there are some patients with lung cancer whose primary focus is very small but have distal metastasis. This seems to indicate that the current cancer treatment guidelines do not fully guide the diagnosis and treatment of cancer. In recent years, with the continuous development of sequencing technology, the concepts of molecular diagnosis and molecular therapy have been deeply rooted in the hearts of the people.^22^ The use of biomarkers for cancer diagnosis and prognosis prediction has gradually become a trend.^20^ Autophagy plays a key role in the occurrence and development of LUAD. Therefore, it is necessary to use autophagy‐related gene set to predict the prognosis of LUAD. Non‐coding RNA plays a key role in the occurrence and development of cancer, so it is of clinical significance to use autophagy‐related non‐coding RNA to predict the prognosis of LUAD. To our knowledge, this study is the first to use autophagy‐associated lncRNA to predict the prognosis of LUAD.

According to the results of the ROC curve, the AUC of the risk score established by 11 autophagy‐related lncRNAs was 0.809, while the AUC of the staging index of the guidelines was only 0.713. This result showed that the risk score established by these 11 autophagy‐related lncRNAs was superior to the current guidelines for predicting prognosis. More interestingly, the AUC of M stage in TNM staging in the current guidelines was only 0.503, which indicated that the prediction of prognosis of distal metastasis in the guidelines may not be accurate. In GSEA, the differential genes of high‐risk group and low‐risk group were enriched in regulation of cell cycle G2 M phase transition, cell cycle G2 M phase transition, ephrin receptor signalling pathway, cell cycle, mismatch repair and proteasome. The results showed that there was a difference in gene expression between the high‐risk group and the low‐risk group, and the risk scores of 11 autophagy‐related lncRNAs were associated with the occurrence and development of LUAD. On the whole, the risk score we established was a quite reliable indicator for predicting the prognosis of LUAD.

So far, the focus of accurate genomic medicine was to find accurate and specific predictors of survival and prognosis from large medical data with clinical results. Therefore, in recent years, there were some studies aimed at using bioinformatics analysis to explore the prognostic factors related to autophagy.^23^, ^24^, ^25^ In the past year, three prognostic risk models of autophagy‐related genes in LUAD were established based on TCGA database and using different screening criteria and statistical methods.^26^, ^27^, ^28^ At the same time, because of the important role of lncRNA in autophagy, the role of lncRNA related to autophagy in cancer had attracted more attention. Recently, autophagy‐related lncRNA prognostic risk models have been established for several cancers, including breast cancer. Therefore, we conducted this study and found a new 11 lncRNA prognostic risk models associated with autophagy, which may help clinicians to make individual and effective treatment decisions.

However, our research also has the following two limitations. First of all, we used traditional statistical analysis methods to establish and evaluate the prognostic risk models of 11 autophagy‐related lncRNAs. Although these methods have been applied and verified in many studies, more advanced methods and technologies are needed to improve our further research in the future. Secondly, in order to further verify our bioinformatics prediction results, 11 lncRNAs related to autophagy need to be further studied, including functional experiments and molecular mechanisms.

## 5 CONCLUSION

In conclusion, we identified a novel autophagy‐related survival model consisting of 11 lncRNAs (GAS6‐AS1, AC106047.1, AC010980.2, AL034397.3, NKILA, AL606489.1, HLA‐DQB1‐AS1, LINC01116, LINC01806, FAM83A‐AS1, and AC090559.1) in LUAD. In the future, these 11 autophagy‐related lncRNAs may become new targets for the treatment of LUAD and provide a more individualized and accurate prognostic monitoring tool.

## CONFLICT OF INTEREST

The authors declare that they have no competing interests.

## AUTHOR CONTRIBUTIONS

**Liwei Wu:** Formal analysis (lead); Methodology (lead); Writing‐original draft (lead). **Zilu Wen:** Supervision (equal); Writing‐original draft (equal). **Yanzheng Song:** Funding acquisition (equal); Writing‐review & editing (equal). **Lin Wang:** Funding acquisition (equal); Writing‐review & editing (equal).

## ETHICAL APPROVAL

Not applicable.

## Data Availability

All data used in this study were acquired from The Cancer Genome Atlas (TCGA) portal.
